# Short-term serial circulating tumor DNA assessment predicts therapeutic efficacy for patients with advanced pancreatic cancer

**DOI:** 10.1007/s00432-023-05594-1

**Published:** 2024-01-26

**Authors:** Hideki Motobayashi, Yuji Kitahata, Ken-ichi Okada, Motoki Miyazawa, Masaki Ueno, Shinya Hayami, Atsushi Miyamoto, Atsushi Shimizu, Masatoshi Sato, Tomohiro Yoshimura, Yuki Nakamura, Norio Takemoto, Tomoki Nakai, Takahiko Hyo, Kyohei Matsumoto, Hiroki Yamaue, Manabu Kawai

**Affiliations:** 1 Second Department of Surgery, School of Medicine, Wakayama Medical University, 811-1 Kimiidera, Wakayama, 641-8510 Japan; 2 Department of Cancer Immunology, Wakayama Medical University, Wakayama, Japan

**Keywords:** Advanced pancreatic cancer, Systemic chemotherapy, Therapeutic efficacy, Real-time biomarkers, Molecular response

## Abstract

**Purpose:**

We investigated the potential clinical utility of short-term serial *KRAS*-mutated circulating cell-free tumor DNA (ctDNA) assessment for predicting therapeutic response in patients undergoing first-line chemotherapy for advanced pancreatic cancer.

**Methods:**

We collected 144 blood samples from 18 patients with locally advanced or metastatic cancer that were undergoing initial first-line chemotherapy of gemcitabine plus nab-paclitaxel (GEM plus nab-PTX). Analysis of *KRAS*-mutated ctDNA was quantified by digital droplet polymerase chain reaction (ddPCR) as mutant allele frequency (MAF). This study investigated pretreatment *KRAS*-mutated ctDNA status and ctDNA kinetics every few days (days 1, 3, 5 and 7) after initiation of chemotherapy and their potential as predictive indicators.

**Results:**

Of the 18 enrolled patients, an increase in *KRAS*-mutated ctDNA MAF values from day 0–7 after initiation of chemotherapy was significantly associated with disease progression (*P* < 0.001). Meanwhile, positive pretreatment ctDNA status (MAF ≥ 0.02%) (*P* = 0.585) and carbohydrate antigen 19-9 (CA19-9) values above the median (*P* = 0.266) were not associated with disease progression. In univariate analysis, this short-term increase in ctDNA MAF values (day 0–7) was found to be associated with significantly shorter progression free survival (PFS) (hazard ration [HR], 24.234; range, (2.761–212.686); *P* = 0.0002).

**Conclusion:**

This short-term ctDNA kinetics assessment may provide predictive information to reflect real-time therapeutic response and lead to effective refinement of regimen in patients with advanced pancreatic cancer undergoing systemic chemotherapy.

**Supplementary Information:**

The online version contains supplementary material available at 10.1007/s00432-023-05594-1.

## Introduction

Patients with unresectable advanced pancreatic cancer have poor survival prognosis (median 5–9 months), which is even worse than that for patients with potentially resectable pancreatic cancer (Cabasag et al. 2022; Kamisawa et al. 2016; Shibuki et al. 2022). Gemcitabine plus nab-paclitaxel (GEM plus nab-PTX) and FOLFIRINOX are the standard chemotherapy regimens recommended for first-line treatment of locally advanced and metastatic pancreatic cancer, and these regimens have provided favorable survival benefits (Conroy et al. 2011, 2018; Von Hoff et al. 2013). However, patients’ responses to chemotherapy are highly divergent. The paucity of reliable predictive biomarkers that accurately reflect therapeutic efficacy in individual patients has been an obstacle in advanced pancreatic cancer treatment. Useful biomarkers to predict prognosis, for personalized treatment, and for monitoring of therapeutic response, are urgently required.

Carbohydrate antigen 19-9 (CA19-9) is a conventional blood-based biomarker recommended for clinical practice by the National Comprehensive Cancer Network (NCCN) guidelines, and an established diagnostic and prognostic indicator for patients with pancreatic cancer (Tempero et al. 2021). However, CA19-9 values are highly affected by concomitant inflammation such as cholangitis, or obstructive jaundice, and these conditions are commonly observed in patients with advanced pancreatic cancer (Cheng et al. 2017; Marrelli et al. 2009). Moreover, approximately 10–22% of patients lack either the Lewis gene or the secretory genes and will not have elevated CA19-9 values in the presence of pancreatic cancer (Tsai et al. 2020). Furthermore, the usefulness of CA19-9 as a reliable marker to assess treatment response in patients undergoing systemic chemotherapy is still unclear (Boeck et al. 2010; Reni et al. 2009).

Liquid biopsy has recently attracted attention as a promising minimally-invasive option that utilizes samples extracted from body fluid, such as peripheral blood and urine. It has been reported that activating *KRAS* mutation are found in more than 90% of cases of pancreatic ductal adenocarcinoma and accumulate at codon 12 and 13, therefore, tumor-derived DNA circulating from primary tumor tissue into the bloodstream are of interest as a surrogate marker (Bailey et al. 2016; Sugimori et al. 2020; Witkiewicz et al. 2015). Plasma-derived circulating cell-free tumor DNA (ctDNA) is shed from tumor cells at primary tumor tissue and metastatic lesions and generated by lysis of tumor cells undergoing apoptosis, necrosis, or proliferation (Liberko et al. 2021). Owing to the nature of its cellular turnover, the mean half-life of ctDNA is quite short at 16.3 min (range, 4–30 min) (Lo et al. 1999), and is therefore suitable for real-time evaluation. On the clinical significance of ctDNA, the amount of ctDNA in plasma reflects tumor tissue burden and the degree of progression, and thus it may be an indicator of oncological malignancy (Siravegna et al. 2019; Strijker et al. 2020). However, whether only a single point assessment of ctDNA values and the genomic features prior to treatment are sufficient to predict subsequent disease prognosis including chemotherapeutic efficacy remains controversial (Zhou et al. 2021). Given the short half-life nature of ctDNA and its molecular biological feature of being derived from dying cells, we proposed that the assessment of short-term kinetics in ctDNA values is crucial to evaluate the chemotherapeutic efficacy in individual patients. Early biomarkers for therapeutic response might lead to early therapeutic intervention (i.e., changes in chemotherapy regimen) in patients who are not expected to respond.

The present study aims to elucidate the predictive value of short-term serial quantitative assessment of *KRAS*-mutated ctDNA values at initial first-line chemotherapy to identify a reliable indicator of therapeutic response in patients with treatment-resistant advanced pancreatic cancer.

## Materials and methods

### Study design and participants

We prospectively enrolled 20 patients with a diagnosis of locally advanced or metastatic pancreatic cancer at the Second Department of Surgery, Wakayama Medical University Hospital (WMUH) between July 2020 and March 2021. Locally advanced or metastatic pancreatic cancer was diagnosed according to the NCCN practice guidelines (version 2, 2021) by radiological confirmation or surgical findings. All participating patients were assessed for presence of distant metastasis by PET-CT prior to treatment. Invasive ductal adenocarcinoma was diagnosed for all eligible patients by pathologic examination with endoscopic ultrasound-fine needle aspiration (EUS-FNA) before treatment initiation. Regarding CA19-9, after administering jaundice-reducing treatment to all cases and observing the disappearance of symptoms related to bile duct inflammation, we proceeded to measure CA19-9 values. This prospective study protocol was approved by the WMUH Institutional Review Board (approval no. 2855) and was registered in the UMIN Clinical Trial Registry (UMIN000041261). All research was performed in accordance with relevant guidelines and regulations. Written informed consent was obtained from all study participants before enrollment.

### Assessment of therapeutic response

The follow-up of the patients and tumor assessment was carried out using computed tomography (CT) or magnetic resonance imaging (MRI) every 8–12 weeks. The CT or MRI scan was independently interpreted by two or more experienced physicians, including hepatobiliary and pancreatic surgeons and radiologists. The interpretations were conducted without knowledge of the results of *KRAS*-mutated status and ctDNA kinetics for each patient, ensuring a blinded approach to the assessment of disease progression. They had consensus on the definition of progression according to Response Evaluation Criteria in Solid Tumors (RECIST) 1.1 criteria for CT imaging. Patient characteristics were retrieved from medical records by a trained medical doctor and analyzed for this study. They included age, gender, body mass index (BMI), tumor size of primary lesion, obstructive jaundice, preoperative serum tumor markers (CA19-9, carcinoembryonic antigen [CEA] values), and positron emission tomography (PET)-computed tomography maximal standardized uptake value (SUV_max_).

### Systemic chemotherapy

All study participants underwent GEM plus nab-PTX as first-line chemotherapy based on a diagnosis of locally advanced or metastatic pancreatic cancer. The GEM plus nab-PTX therapy comprised a 30-min intravenous infusion of nab-paclitaxel at a dose of 125 mg/m^2^, followed by a 30-min intravenous infusion of gemcitabine at a dose of 1000 mg/m^2^ on days 1, 8, and 15 during a four-week period as one cycle of regimen. We adhered to the prescribed regimen, as the initial administration was for all patients with PS 0–1.

### Blood sample collection and extraction of cell-free DNA

The blood sample collection and extraction of cell-free DNA (cfDNA) were performed at serial points; the day before initiation of chemotherapy (pretreatment, day 0), and 1, 3, 5, 7 days after initiation of the first-line chemotherapy, and additional longitudinal samples were obtained during follow-up. The whole peripheral blood samples (7 ml) were collected in ethylenediaminetetraacetic acid (EDTA) tubes from each patient and centrifuged at 1900 g for 10 min at 4 °C for plasma separation within 2 h of blood collection. The plasma was then transferred into 1.5 ml Eppendorf tubes, then centrifuged at 16,000 g for 10 min at 4 °C. The supernatant plasma samples were separated and stored at − 80 °C until cfDNA extraction. The process of cfDNA purification was the same as in our previous study (Kitahata et al. 2022; Nakamura et al. 2021).

Next, cfDNA was extracted from 3 mL of plasma using the QIAamp Circulating Nucleic Acid Kit (Qiagen) according to the manufacturer’s instructions. Samples were eluted in 75 µL of elution buffer, and cfDNA was frozen at – 80 °C until subsequent use.

### Quantification of KRAS-mutated ctDNA by digital droplet PCR (ddPCR) analysis

Analysis of *KRAS* somatic mutations in cfDNA was quantified by digital droplet polymerase chain reaction (ddPCR) using the QX200 Droplet Digital PCR System (Bio-Rad Laboratories) and ddPCR *KRAS* multiplex assays including G12A, G12C, G12D, G12R, G12S, G12V, G13D mutant codons (Bio-Rad Laboratories) according to the manufacturer’s protocols, as previously described (Kitahata et al. 2022; Nakamura et al. 2021). A reaction volume of 20 µL, including 8 µL of cfDNA, was used as a template for each PCR. Droplets were generated using the QX200 droplet generator (Bio-Rad Laboratories), and PCR reaction was performed in a C1000 Touch Thermal Cycler (Bio-Rad Laboratories) under the following conditions: 95 °C for 10 min, 40 cycles of 94 °C for 30 s, 55 °C for 1 min, and 98 °C for 10 min. Data analyses were performed using QuantaSoft software version 1.7.4 (Bio-Rad Laboratories), including the calculation of the fractional abundance. *KRAS*-mutated ctDNA values were quantitatively compared by the index of mutant allele frequency (MAF) value. The MAF per reaction is calculated as a percentage (%) of the (number of total mutated copies) / (number of total mutated copies + number of total wild-type copies). ctDNA positivity was defined as a lower limit for MAF at 0.02% according to our previous report (Kitahata et al. 2022; Nakamura et al. 2021). ddPCR analysis was performed without clinical information such as therapeutic efficacy and disease progression. Each sample undergoes analysis twice, and in cases of discordant results, a third analysis is performed. Subsequently, thorough data scrutiny is conducted collaboratively with co-authors to ensure the most accurate values are detected. This approach has been employed to address concerns related to the possibility of false negatives due to low cfDNA input.

### Statistical analysis

All statistical analyses were performed using JMP version 14.1.0 (SAS institute). To compare the difference in means of MAF value between the two groups, we used Mann–Whitney (rank-sum) test and Wilcoxon signed rank test (non-normally distributed continuous variables). Differences between groups were determined using Pearson’s chi-square test or the Fisher’s exact test to compare categorical clinical characteristics as appropriate. Progression-free survival (PFS) was defined as the time from the start of chemotherapy to the first radiological evidence of progression according to RECIST 1.1 criteria. Patients who did not progress during the follow-up period were censored. The Kaplan–Meier method with log-rank test was used to determine the statistical significance. Univariate analysis was performed using the Cox proportional hazards regression model to examine whether the different variables were associated with PFS. Multivariable analysis was performed with multiple logistic regression to identify independent risk factors of PFS. These analyses indicated that an odds ratio with a 95% confidence interval (CI) was calculated for each factor. A *P*-value of ≤ 0.05 was considered statistically significant.

### Data availability

The processed data generated in this study are available within the article and its supplementary data files. Due to the sensitive nature of the data, information analyzed during the present study is available from the corresponding author upon reasonable request.

## Results

### Patient characteristics

**Fig. 1:**
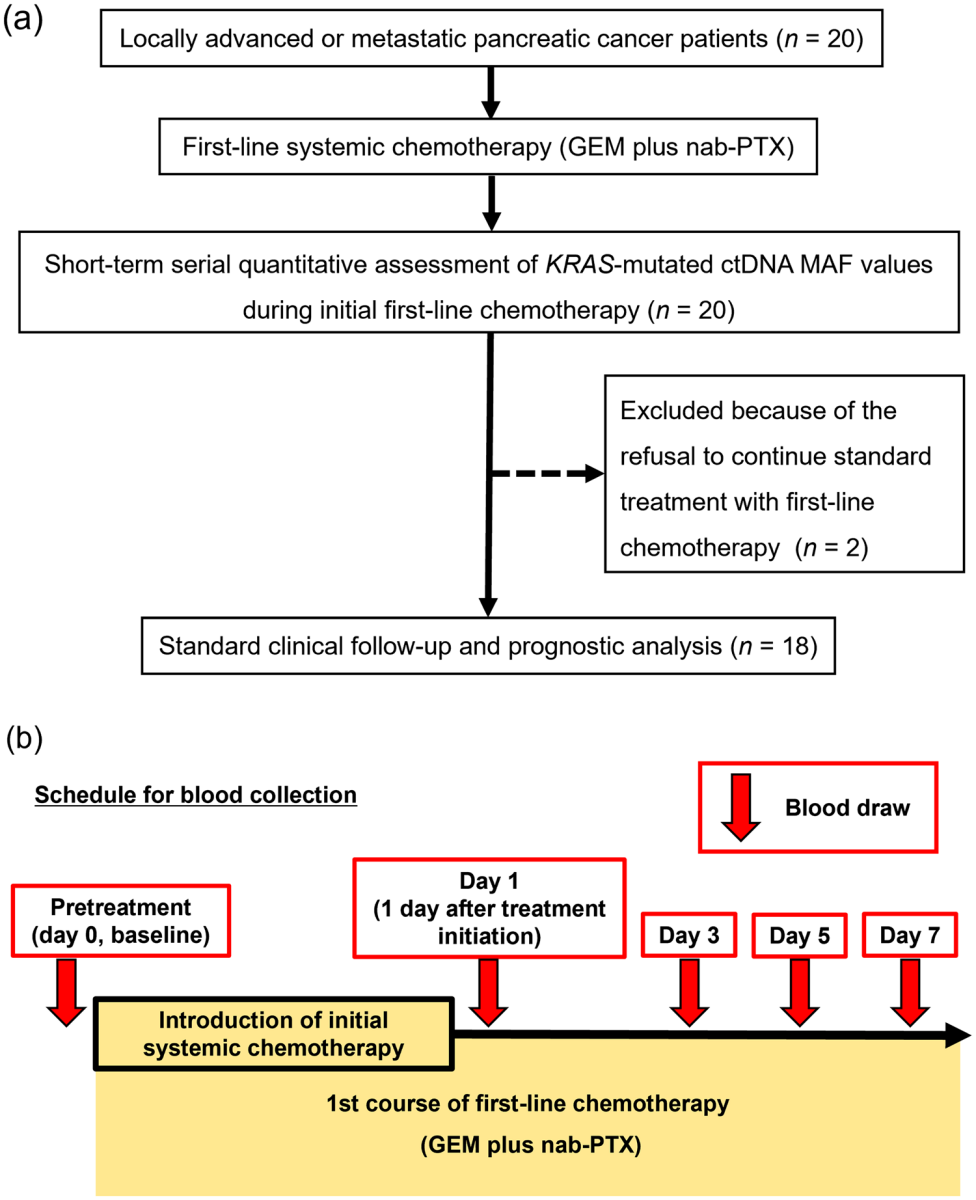
**a** Consort diagram of patient enrollment and exclusion. Patient enrollment, sample collection and analysis, and evaluable population; **b** Study schema of short-term serial measurements for patients with locally advanced or metastatic pancreatic cancer undergoing initial first-line chemotherapy (GEM plus nab-PTX). GEM plus nab-PTX, Gemcitabine plus nab-paclitaxel; ctDNA, circulating cell-free tumor DNA; MAF, mutant allele frequency

The study recruited and enrolled 20 treatment-naïve patients with locally advanced or metastatic pancreatic cancer who consented to inclusion. All participating patients in our study had Performance Status of 0–1. All 20 patients had short-term serial ctDNA measurements during the initial first-line chemotherapy (GEM plus nab-PTX) and their quantitative changes in *KRAS*-mutated ctDNA MAF values were analyzed. Subsequently, two patients were excluded from our analysis because of their refusal to continue standard treatment regimens with first-line chemotherapy. Finally, 18 patients were analyzed for the short-term serial ctDNA study, and 144 blood samples including follow-up samples from 18 patients were analyzed for ctDNA quantitative assessment (Fig. 1, panel a).

In our cohort, 14 patients (77.8%) presented with locally advanced pancreatic cancer and four with metastatic pancreatic cancer (22.2%) (Table 1). Of the four patients with metastatic pancreatic cancer, one patient (5.6%) presented with liver metastases, one patient with liver and lung metastases (5.6%), one patient with distant lymph node metastases (5.6%), and one patient with peritoneal dissemination (detected during surgical exploration) (5.6%). The median tumor size of the primary lesion at pretreatment was 27 mm (range, 20–50). CA19-9 value analysis were available at pretreatment in all 18 patients, with the median CA19-9 of 363.5 U/ml (range, 25.8–2963 U/ml) and the median CEA value was 6.0 ng/ml (range, 1.4–31.3 ng/ml) at pretreatment. The median PET SUVmax at pretreatment was 6.51 (range, 2.41–11.93). Regarding the presence of *KRAS* mutation within tumor tissues in all 18 patients, only 12 patients in which sufficient specimens could be obtained by fine needle aspiration (FNA) biopsy and one patient with a peritoneal dissemination specimen revealed *KRAS* codon 12/13 mutations within tumor tissue samples. The other five patients did not undergo FNA biopsy or underwent FNA biopsy, but only unqualified tissue specimens could be obtained. It was, therefore, not possible to analyze the presence of genetic mutations within tumor tissue samples from these five cases (Supplementary Table S1).

**Table 1:** Clinical characteristics of patients with pancreatic cancer enrolled at study entry (pretreatment)

Clinical characteristics	*n* = 18
Age, median (range), years	71.5 (51–79)
Gender (male/female), *n*	11/7
BMI, median (range), kg/m^2^	20.915 (16.87–28.49)
Tumor size of primary lesion (pretreatment), median (range), mm	27 (20–50)
Pretreatment positive ctDNA status, *n* (%)	11 (61.1%)
CA19-9 (pretreatment), median (range), U/ml	363.5 (25.8–2963)
CEA (pretreatment), median (range), ng/ml	6.0 (1.4–31.3)
PET SUV_max_ (pretreatment), median (range)	6.510 (2.46–11.93)
Locally advanced/metastatic, *n*	14/4
Obstructive jaundice, *n* (%)	5 (27.8%)

*BMI* body mass index; *ctDNA* circulating cell-free tumor DNA; *CA19-9* carbohydrate antigen 19-9; *CEA* carcinoembryonic antigen; *PET* positron emission tomography; *SUV*_*max*_ maximal standardized uptake value

Of the 18 patients included in this study, *KRAS*-mutated ctDNA was detected (positive, MAF ≥ 0.02%) in 11 patients (*n* = 11/18, 61.1%) at pretreatment. The clinical characteristics of the 18 patients are summarized in Table 1. Regarding the classification of locally advanced pancreatic cancer (*n* = 14) or metastatic pancreatic cancer (*n* = 4), there was no difference between the two groups in *KRAS*-mutated ctDNA MAF values at pretreatment (Mann–Whitney *U*-test, *P* = 0.7018) (Supplementary Fig. S1).

### Prognostic value of pretreatment KRAS-mutated ctDNA status and CA19-9 values

Median follow-up time for all patients was 365 days (range, 111–475 days). Overall, eight patients (44.4%) had radiological progression. There was no significant difference in baseline pretreatment *KRAS*-mutated ctDNA MAF values between the patients with disease progression (*n* = 8) and those without progression (*n* = 10) (Mann–Whitney *U*-test, *P* = 0.3365) (Supplementary Fig. S2). Regarding prognostic analysis of disease progression, the patients with positive pretreatment *KRAS*-mutated ctDNA status had no significant decrease in median PFS (391 days; 4 of 11 patients [36.4%] had progression disease) compared with those with negative ctDNA status (PFS: 301 days; 4 of 7 [57.1%] patients had disease progression; *P* = 0.585; Supplementary Fig. S3, panel a). Meanwhile, the median CA19-9 value was 363.5 U/ml, and pretreatment CA19-9 values were not associated with statistically significant poor PFS between the patients with CA19-9 values higher than 363.5 U/ml (median) and those with CA19-9 values lower (PFS: 301 vs. 374 days; *P* = 0.266; Supplementary Fig. S3, panel b).

### Short-term serial ctDNA assessment as a reliable predictor for progression disease

**Fig. 2:**
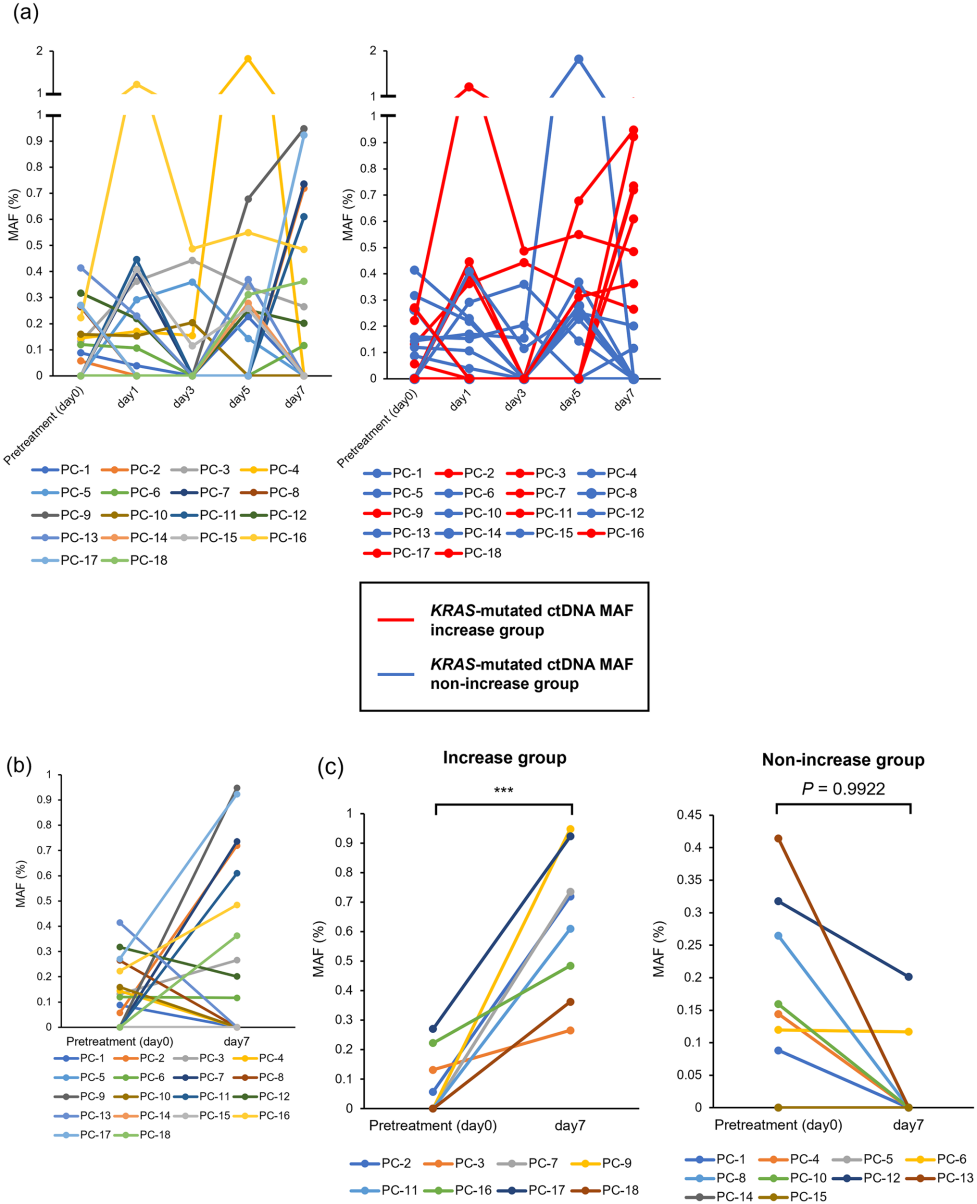
Absolute *KRAS*-mutated ctDNA MAF kinetics from day 0 to 7 after initial first-line chemotherapy. **a** Absolute ctDNA MAF kinetics on each day until day 7. The patients in the increase group are shown in red, and those in the non-increase group are shown in blue from day 0 to 7; **b** MAF kinetics between two time points (day 0 and 7); **c** Patients with increased MAF from day 0 to 7 were classified as the increase group, and those without increased MAF were classified as the non-increase group. The kinetics of MAF between day 0 and day 7 were analyzed in each group. Only the increase group had a significantly different MAF variation, by Wilcoxon signed rank test. *P < 0.05, **P < 0.01, ***P < 0.001. ctDNA, circulating cell-free tumor DNA; MAF, mutant allele frequency

As noted above, all 18 patients underwent GEM plus nab-PTX as first-line chemotherapy for locally advanced or metastatic pancreatic cancer. Blood *KRAS*-mutated ctDNA MAFs were measured before chemotherapy (pretreatment, day 0), and on days 1, 3, 5 and 7 after initiation of the first-line chemotherapy (Fig. 1, panel b), and these short-term serial *KRAS*-mutated ctDNA kinetics were evaluated in each patient. The various detailed ctDNA kinetics in each patient are depicted in Supplementary Fig. S4, panel a and b. In light of these individual ctDNA kinetics, we focused on the short-term quantitative change in *KRAS*-mutated ctDNA MAF values between pretreatment (day 0, baseline) and day 7 after chemotherapy initiation, which we considered to be a potential early prognostic indicator of the therapeutic response and efficacy for chemotherapy. To investigate this hypothesis, patients were classified into two groups based on the short-term quantitative kinetics in absolute *KRAS*-mutated ctDNA MAF values from day 0 to 7 (Fig. 2, panel a, b and c). Thus, cases in which ctDNA MAF values (%) increased even slightly from day 0 to 7 were defined as the increase group, while those with no increase, including those with no observable change, were defined as the non-increase group (Fig. 2, panel c). In addition, we analyzed statistically whether the change from day 0 to 7 was significant in increase and non-increase groups, respectively, using the Wilcoxon signed rank test. As a result, only the increase group showed significantly different changes in *KRAS*-mutated ctDNA MAF values between day 0 and 7 (Wilcoxon signed rank test, *P* = 0.0039), while the non-increase group showed no significant difference (Fig. 2, panel c). The patients’ clinical characteristics were summarized at the time of diagnosis according to two different ctDNA MAF kinetics classification (Table 2). We analyzed age, gender, BMI, radiologic tumor size of primary lesion, CA19-9, CEA, PET SUV_max_, locally advanced or metastatic, and obstructive jaundice. Although CEA values were significantly higher in the increase group and more patients with metastatic pancreatic cancer were included in the non-increase group, other features were not significantly different between these two groups (Table 2).

**Table 2:** Clinical characteristics according to ctDNA kinetics status, ctDNA MAF increase group versus non-increase groups

Clinical characteristics	Short-term ctDNA MAF kinetics status		*P*-value
	Increase group (*n* = 8)	Non-increase group (*n* = 10)	
Age, median (range), years	71.5 (65–79)	73 (55–79)	0.6814
Gender (male/female), *n*	5/3	6/4	0.9139
BMI, median (range), kg/m^2^	18.04 (16.87–24.22)	21.28 (17.07–28.49)	0.3428
Tumor size of primary lesion (pretreatment), median (range), mm	29 (24–50)	25.5 (20–40)	0.3428
CA19-9 (pretreatment), median (range), U/ml	536.5 (81–2963)	252.1 (25.8–2026)	0.6814
CEA (pretreatment), median (range), ng/ml	13.65 (4.3–31.3)	3.6 (1.4–15.5)	**0.0044**
PET SUV_max_ (pretreatment), median (range)	6.62 (5.14–11.93)	5.4 (2.46–6.95)	0.1888
Locally advanced/metastatic, *n*	8/0	6/4	**0.0425**
Obstructive jaundice, *n*	2	3	0.8139

Boldface font indicates statistically significant *P*-value

*ctDNA* circulating cell-free tumor DNA; *MAF* mutant allele frequency; *BMI* body mass index; *CA19-9* carbohydrate antigen 19-9; *CEA* carcinoembryonic antigen; *PET* positron emission tomography; *SUV*_*max*_ maximal standardized uptake value

**Fig. 3:**
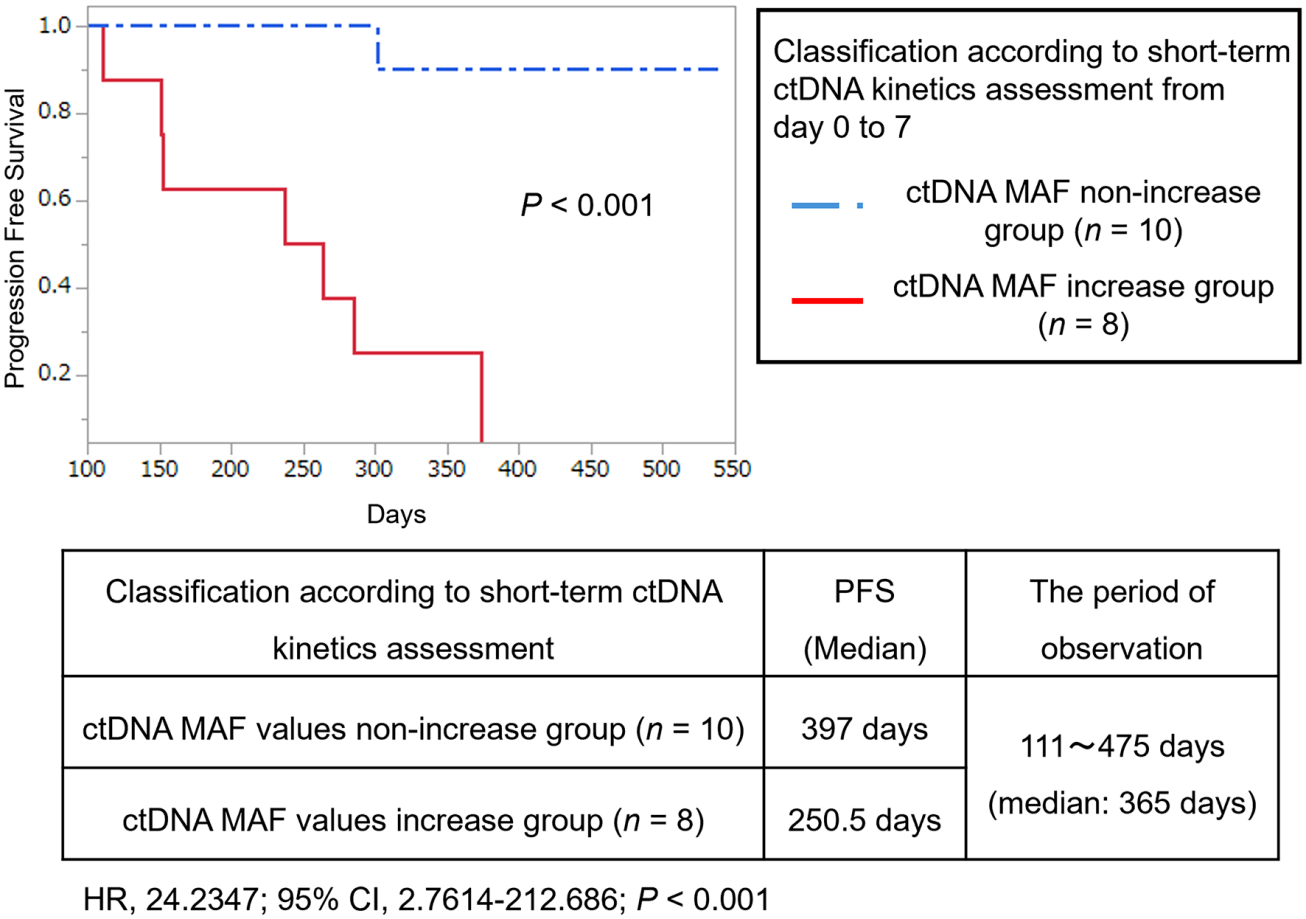
Kaplan–Meier curves of progression-free survival (PFS) classified according to short-term ctDNA kinetics assessment from day 0 to 7 after initial first-line chemotherapy. The patients with increased *KRAS*-mutated ctDNA MAF from day 0 to 7 had significant decrease in median PFS (250.5 days; 7 of 8 patients [87.5%] had progression) compared with the patients with non-increase *KRAS*-mutated ctDNA MAF (PFS: 397 days; 1 of 10 patients [10%] had progression; *P* < 0.001). ctDNA, circulating cell-free tumor DNA; MAF, mutant allele frequency; HR, hazard ratio; CI, confidence interval

**Fig. 4:**
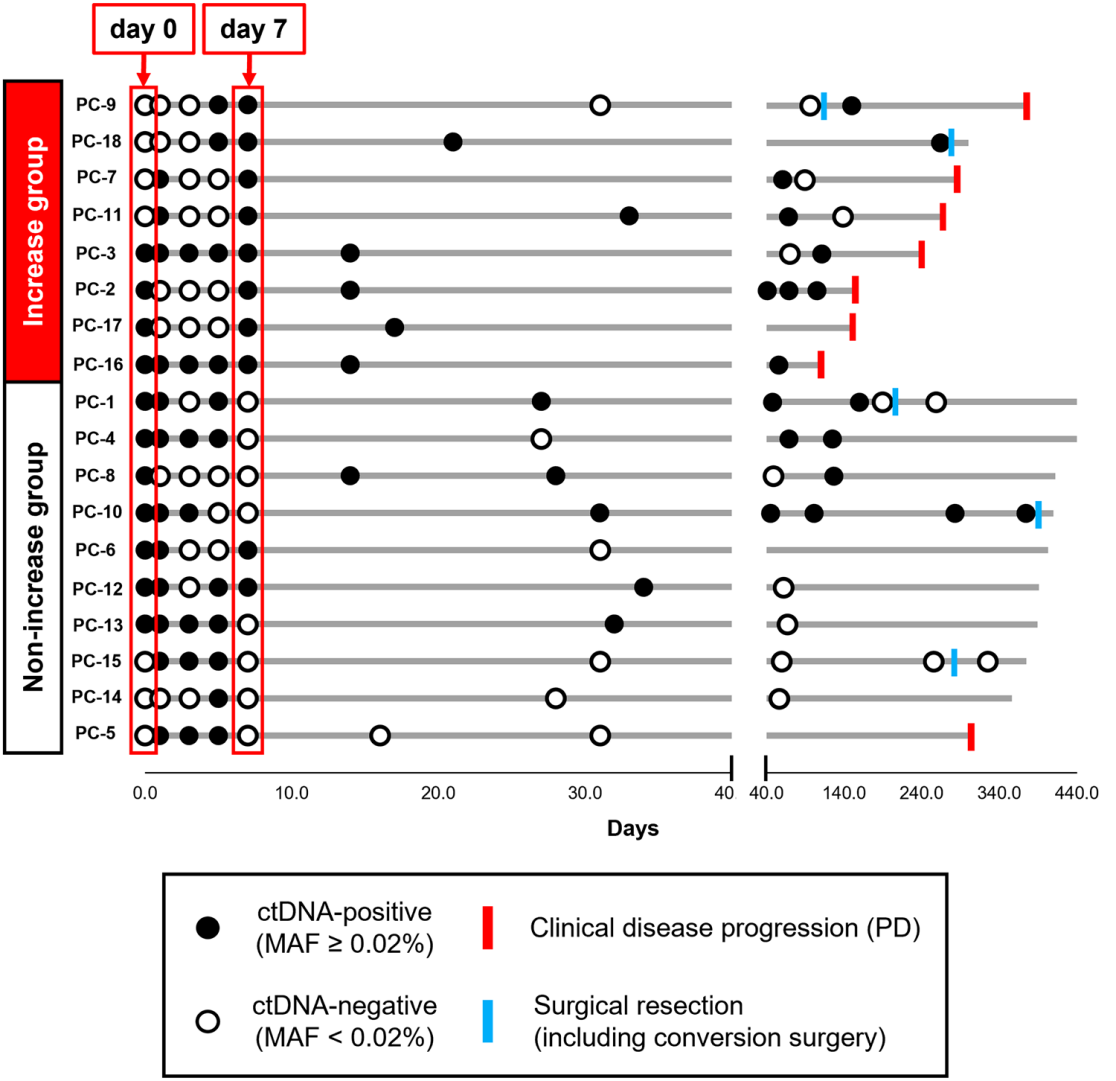
Swimmer plots of 18 patients with locally advanced or metastatic pancreatic cancer undergoing first-line chemotherapy with GEM plus nab-PTX. Patients whose ctDNA MAF values increased from day 0 to 7 (increase group) are shown at the upper part of the figure, and those whose ctDNA MAF values did not increase (non-increase group) are shown at the lower part of the figure. Filled black circles, ctDNA-positive (*KRAS*-mutated ctDNA were detected); unfilled black circles, ctDNA-negative (*KRAS*-mutated ctDNA were not detected). Red bars are the times of RECIST progressive disease (PD) due to enlargement of the primary lesion and/or to appearance or enlargement of distant metastasis. Blue bars are the times of surgical resection including conversion surgery. GEM, gemcitabine; nab-PTX, nab-paclitaxel; ctDNA, circulating cell-free tumor DNA; MAF, mutant allele frequency

As a result, the patients with increased *KRAS*-mutated ctDNA MAF values from day 0 to 7 had a significant decrease in median PFS (250.5 days; 7 of 8 patients [87.5%] had progression) compared with the patients with non-increase *KRAS*-mutated ctDNA MAF values (PFS: 397 days; 1 of 10 patients [10%] had progression; *P* < 0.001; Fig. 3). The longitudinal ctDNA monitoring data after the initial first-line chemotherapy for each patient were represented in swimmer plots (Fig. 4). Most patients in the increase group had disease progression during the observation period, while most patients in the non-increase group were able to continue systemic chemotherapy without disease progression. Surgical resection including conversion surgery was conducted in two patients (PC-9: Locally advanced PC, PC-18: Locally advanced PC) in the increase group and in three patients (PC-1: Locally advanced PC, PC-10: Metastatic PC, PC-15: Locally advanced PC) in the non-increase group (Fig. 4).

### Univariate and multivariate analysis of patient outcomes

In univariable analysis, an increase in absolute *KRAS*-mutated ctDNA MAF values (day 0–7) at initial chemotherapy and CEA value ≥ 6 (median value) were found to be associated with significantly shorter PFS (hazard ratio [HR], 24.2347; range, (2.761–212.686); *P* = 0.0002; HR, 3.5721e^9^; *P* < 0.0001, Table 3). Other clinical features (gender, age, tumor size of primary lesion, pretreatment *KRAS*-mutated ctDNA status and CA19-9 values) were not associated with PFS in this study.

**Table 3:** Univariable Cox regression analysis predicting progression-free survival (PFS) in 18 patients with locally advanced or metastatic pancreatic cancer

Variables	Univariate analysis		Multivariate analysis	
Clinical characteristics	HR (95% CI)	*P*-value	HR (95% CI)	*P*-value
*Gender*				
Male/female	0.5917 (0.1473–2.3758)	0.4619		
*Age*				
≥ 72/ < 72	0.8447 (0.2110–3.3816)	0.8116		
*Tumor size of primary lesion (pretreatment)*				
≥ 27 mm (median value)/ < 27 mm	3.3553 (0.6699–16.8061)	0.1121		
*Pretreatment KRAS-mutated ctDNA status*				
Positive/negative	0.6772 (0.1657–2.7677)	0.5883		
*ctDNA MAF kinetics status (day 0 to 7)*				
Increase/Non-increase	24.2347 (2.7614–212.686)	**0.0002**	5.1096 (0.5976–43.6871)	0.1363
*Pretreatment CA19-9 values*				
≥ 363.5 (median value)/ < 363.5	2.2135 (0.5260–9.3140)	0.2687		
*Pretreatment CEA values*				
≥ 6.0 (median value)/ < 6.0	3.5721e^9^	** < 0.0001**	1.498e^9^	0.9989

Boldface font indicates statistically significant *P*-value

*HR* hazard ratio; *CI* confidence interval; *ctDNA* circulating cell-free tumor DNA; *MAF* mutant allele frequency; *CA19-9* carbohydrate antigen 19-9; *CEA*, carcinoembryonic antigen

In multivariate analysis, no statistically significant differences were found for both an increase in absolute *KRAS*-mutated ctDNA MAF value (day 0–7) and CEA value ≥ 6 (Table 3).

## Discussion

The current study demonstrated that the short-term on-treatment increase kinetics in *KRAS*-mutated ctDNA MAF values at initial chemotherapy might be a significant predictor for patients with locally advanced or metastatic pancreatic cancer and undergoing first-line systemic chemotherapy. Furthermore, it has been revealed that CEA status serves as a significant prognostic marker. In contrast, pretreatment (day 0, baseline) *KRAS*-mutated ctDNA status was insufficient as a predictive marker, as well as pretreatment (baseline) CA19-9 status and other clinical features in this study. Based on the above results, we focused on the importance of early dynamic kinetics in ctDNA after initiation of systemic chemotherapy, and measured MAF fluctuations every few days in individual patients (Supplementary Fig. S4, panel a and b). As a result, a comparison of ctDNA MAF values between the pretreatment (day 0, baseline) and day 7 of the first cycle of GEM plus nab-PTX might serve as an early indicator of treatment effectiveness. The lack of significance in the multivariable analysis for ctDNA and CEA status might be attributed to a potential correlation between these two variables. Upon conducting a Pearson's correlation analysis, a significant correlation was indeed observed (Fisher’s exact test, *P* = 0.0152). However, we acknowledge that, in terms of the rise in CEA and ctDNA, there may be nuances in their respective clinical significance. We believe that, compared to CEA, ctDNA more dynamically reflects in vivo changes, underscoring the importance of longitudinal analysis. Thus, our study only begins to explore the potential of ctDNA as a liquid biopsy, and we envision that ctDNA, as a novel material, will continue to be a subject of analysis in future studies. Considering the limited sample size in this study, we recognize the need for larger prospective studies to further deepen our understanding based on the insights gained from this investigation.

ctDNA was reported to be a reliable prognostic biomarker for long-term survival in patients with pancreatic cancer (Earl et al. 2015; Hadano et al. 2016), although several other studies suggested that the one time point analysis of the baseline ctDNA status as a clinically significant biomarker has inconsistency regarding its prognostic and predictive potential in patients with pancreatic cancer undergoing multimodality treatment (Allenson et al. 2017; Bernard et al. 2019; Sugimori et al. 2020). Furthermore, whether the baseline ctDNA status and genomic features can predict the tumor sensitivity to chemotherapy before commencement of the treatment remains unverified, although tumor therapeutic response to chemotherapy strongly contributes to prognosis. Therefore, we considered that short-term and serial measurement and assessment for ctDNA of each patient, taking into account the molecular response for chemotherapy to the tumor tissue, would be beneficial as a more sensitive and personalized prognostic indicator. Early response information for the treatment provides more appropriate opportunities for adjusting treatment strategy than conventional tumor markers and radiological imaging. In addition, this strategy would avoid unnecessary toxicities and extra medical expenses to the patients and deterioration of performance status caused by continued exposure to ineffective regimens.

ctDNA is a component of fragmented cfDNA that is derived from tumor cells which have died, mainly due to apoptosis and necrosis. In this context, ctDNA levels are influenced by rates of cell turnover and mechanism of cell death (Sanz-Garcia et al. 2022), and naturally, they are expected to be greatly affected by tumor cell and non-tumor cell death induced by cytotoxic systemic chemotherapy. Also, with particular attention to the in vivo dynamics of ctDNA, one reason that ctDNA is considered to be a sensitive real-time biomarker is that its mean half-life was 16.3 min (range, 4–30 min) (Lo et al. 1999), which is shorter than that of pre-existing protein-based tumor markers. This rapid metabolism and clearance of cfDNA in vivo has been indicated to involve not only renal excretion, but also immune-mediated clearance through the reticuloendothelial system into the liver and spleen (Sanz-Garcia et al. 2022). Given this biology and the short half-life characteristics in ctDNA, it is our belief that early information of tumor-derived ctDNA kinetics on treatment would allow tracking of early therapeutic molecular response to the tumor.

Regarding a previous report which focused on early changes in ctDNA kinetics, Kruger et al. reported that ^mut^*KRAS* ctDNA values increase on or after day 14 from treatment initiation indicated later radiological disease progression (Kruger et al. 2018). ^mut^*KRAS* ctDNA kinetics obtained by serial ctDNA measurements were a more sensitive and highly specific marker of disease progression compared with already established protein-based tumor markers. Similarly, Sugimori et al. reported that patients with locally-advanced or metastatic pancreatic cancer whose *KRAS* mutation in ctDNA remained positive (*n* = 5) after the initial course of chemotherapy had a significantly worse PFS than those whose ctDNA had disappeared (*n* = 8) (Sugimori et al. 2020). They also demonstrated that *KRAS*-MAF continuous assessment might be more useful for monitoring the disease state of *KRAS*-mutated pancreatic ductal adenocarcinoma during chemotherapy. Notably, our results indicated that patients with increased *KRAS*-mutated ctDNA MAF values from day 0 to 7 had a significantly poorer PFS compared with the patients with non-increase *KRAS*-mutated ctDNA MAF values. Furthermore, all participating patients underwent a homogeneous first-line chemotherapy regimen (GEM plus nab-PTX). We also found that ctDNA status as early as day 7 after only the first administered chemotherapy agent might be an early predictor of clinical progression compared with baseline ctDNA status and other conventional protein-based tumor markers. Generally, malignant tumors with distant liver metastases are known to be highly positive for ctDNA in plasma (Newhook et al. 2023). On the other hand, a certain percentage of patients with advanced pancreatic cancer also have negative pre-treatment ctDNA, we validated the significance of short-term and serial measurements even if baseline ctDNA is negative in this study. Consequently, the prognosis for patients with baseline negative ctDNA were PFS 301 days for PC-5 (metastatic), 285 days for PC-7 (locally advanced), 374 days for PC-9 (locally advanced), 264 days for PC-11 (locally advanced), 356 days for PC-14 (locally advanced), 375 days for PC-15 (locally advanced), and 300 days for PC-18 (locally advanced), respectively. The above results indicate that even among baseline ctDNA-negative patients, there are patients with relatively poor or favorable prognosis, thus we still considered it insufficient to evaluate only by a single point of baseline ctDNA. Therefore, we focused on the usefulness of short-term serial assessment of ctDNA. This short-term and early assessment including biological molecular treatment efficacy may, therefore, be beneficial in clinical practice for multimodality treatment.

In this study, the pretreatment CA19-9 status was shown to have no association with disease progression. In our cohort, median CA19-9 was used as the cut-off value for the analysis because CA19-9 values in our cohort were abnormally high in all other patients with one exception due to advanced stage of pancreatic cancer. Nonetheless, an appropriate cut-off value for CA19-9 has not been established as a therapy surveillance in advanced pancreatic cancer, so whether this protein-based tumor marker can be translated into clinical practice as an early indicator for treatment efficacy still requires elucidation. Taken together, easy serial *KRAS*-mutated ctDNA kinetics assessment might provide more precise and preferable information to evaluate oncologic status than CA19-9 values. However, there are many reports about usefulness of CA19-9. We could not conclude the usefulness of CA 19-9 as a prognostic indicator in our study because of the quite limited sample size.

Importantly, genetic analysis of biopsy tissue specimen for *KRAS* mutations was available in only 13 of 18 patients in our study (Supplementary Table S1), and 8 of these cases were positive for ctDNA in the pretreatment, resulting in a concordance rate of 61.5%. There is a report that the profile of *KRAS* mutations in pancreatic cancer tissue is correlate with prognosis (Buscail et al. 2020). On the other hand, it has also been reported that detailed profile of *KRAS* mutations in FNA biopsy specimens is limited (Buscail et al. 2020), where it is difficult to obtain qualified tissue for molecular profiling. Moreover, most tissue samples obtained from advanced pancreatic cancer are only a small fraction derived from fine needle aspirates and may not represent the entire tumoral genetic landscape due to intra-tumor heterogeneity and clonal evolution (Gerlinger et al. 2012). In contrast, liquid biopsy provides information derived from a wide variety of tissues and cells, including the primary tumor and metastatic lesions, which has the potential of overcoming intra-tumor heterogeneity (Wan et al. 2017).

Our study had several serious limitations. First, only an extremely limited number of patients could be included in this analysis. In this study, we focused on the utility of short-term, serial measurements. However, there are no evidence of appropriate intervals for short-term measurements, thus we actually measured every other day and validate ctDNA dynamic kinetics, which resulted in a small sample size because of patient physical burden. We performed exploratory research in a small sample size. We could demonstrate the concept in this issue, so we would like to validate our findings (day 0–7 ctDNA assessment) with large-scale prospective studies and establish more robust evidence in the future. Second, the study was performed at a single institution with an explorative study design during the relatively short period of follow-up. Third, although we focused on ctDNA status on day 7 after treatment initiation, few definite conclusions have been established regarding the optimal sequential timing for blood sampling in other carcinomas and in other chemotherapy regimens, so further validation is required for this issue. Finally, in terms of prognostic analysis, the observation period in this study was relatively short, so prognostic analysis did not include overall survival, only PFS. On this issue, several reports have demonstrated that PFS could be a surrogate indicator for overall survival in advanced pancreatic cancer treated with chemotherapy (Hamada et al. 2016; Makris et al. 2017). However, this study might underline that early information for treatment response based on ctDNA dynamics could offer great clinical utility and lead to new treatment strategy concepts.

In conclusion, our results suggest the potential clinical utility of short-term ctDNA kinetics assessment to provide unique predictive information to dynamically track real-time therapeutic molecular response. This may lead to effective refinement of regimen in patients with advanced pancreatic cancer undergoing systemic first-line chemotherapy, and may aid in anticipating clinical progression. This short-term easy serial evaluation may contribute to optimization of individualized multimodality treatment strategy not only in pancreatic cancer, but also in other cancers. Further large prospective investigations are required to confirm these issues based on our findings.

## Supplementary Information

Below is the link to the electronic supplementary material.

Supplementary file1 (XLSX 11 KB)

Supplementary file2 (TIF 239 KB)

Supplementary file3 (TIF 264 KB)

Supplementary file4 (TIF 556 KB)

Supplementary file5 (TIF 581 KB)

Supplementary file6 (TIF 641 KB)

Supplementary file7 (TIF 883 KB)

## Data Availability

The processed data generated in this study are available within the article and its supplementary data files. Due to the sensitive nature of the data, information analyzed during the present study is available from the corresponding author upon reasonable request.
