# Supplementary figures and images for: Short-term serial circulating tumor DNA assessment predicts therapeutic efficacy for patients with advanced pancreatic cancer
Source: J Cancer Res Clin Oncol. 2024 Jan 26;150(2):35. doi: 10.1007/s00432-023-05594-1 (PMC10817839; doi:10.1007/s00432-023-05594-1)

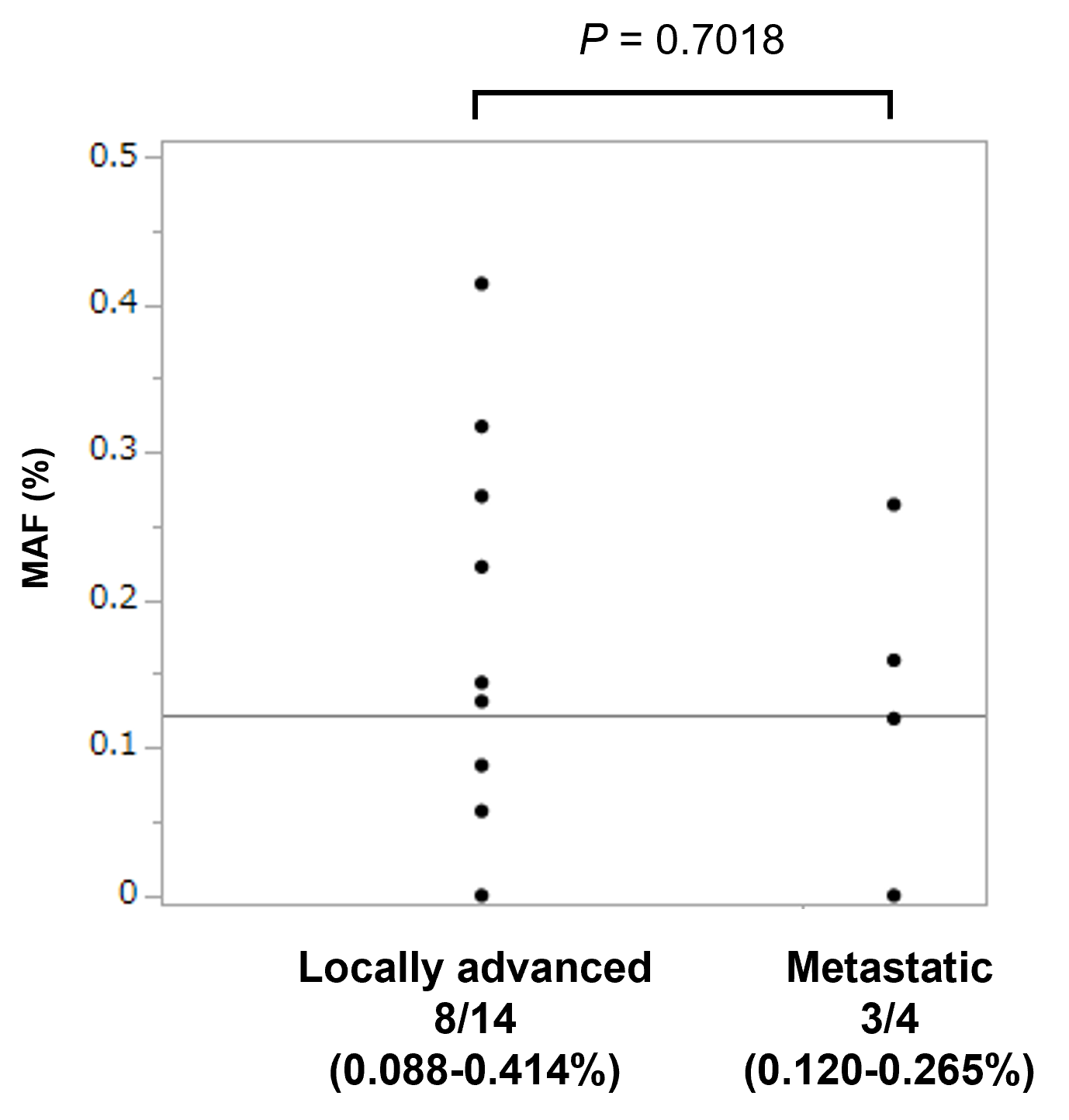

Supplement: Supplementary file 2 — Supplementary file2 (TIF 239 KB) [file 432_2023_5594_MOESM2_ESM.tif]

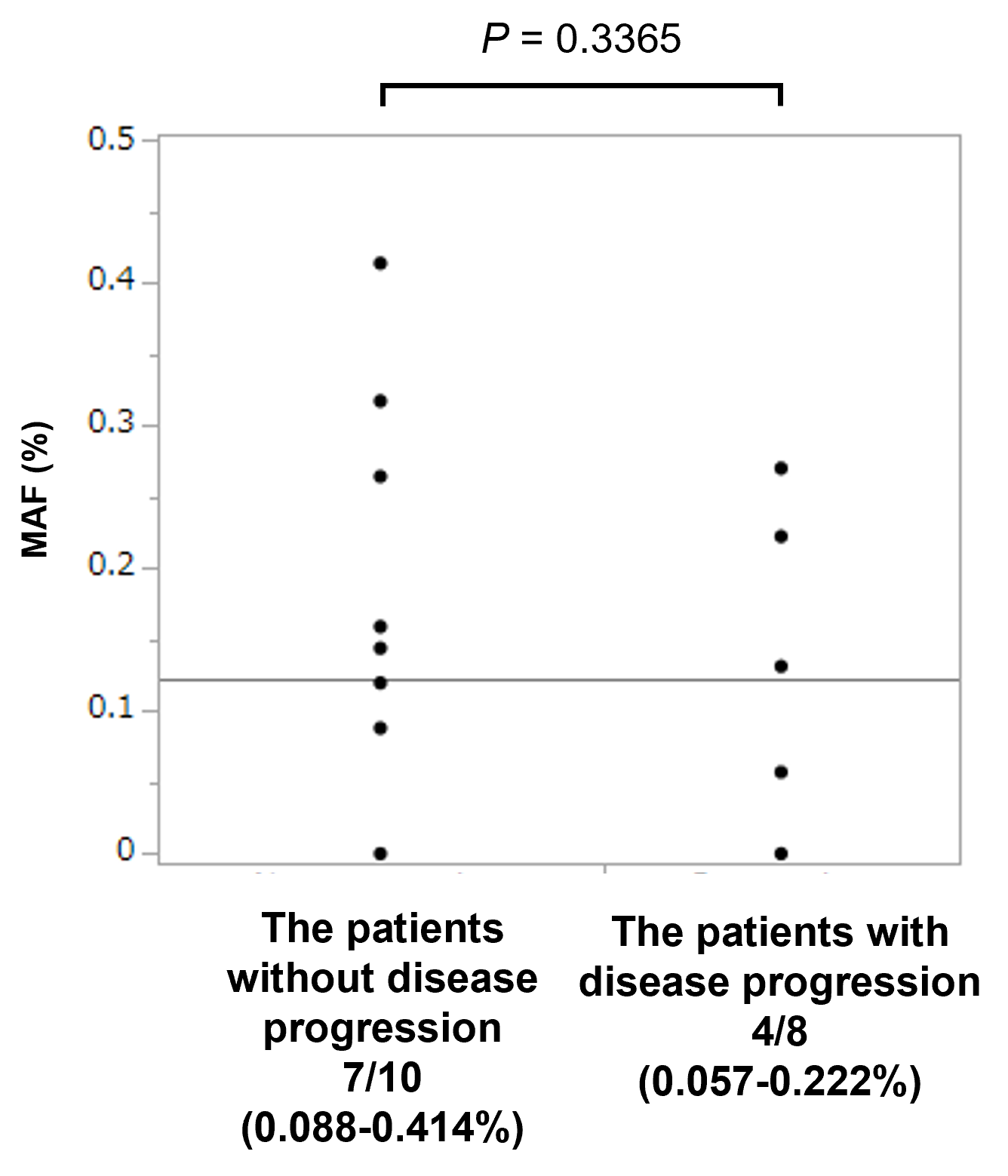

Supplement: Supplementary file 3 — Supplementary file3 (TIF 264 KB) [file 432_2023_5594_MOESM3_ESM.tif]

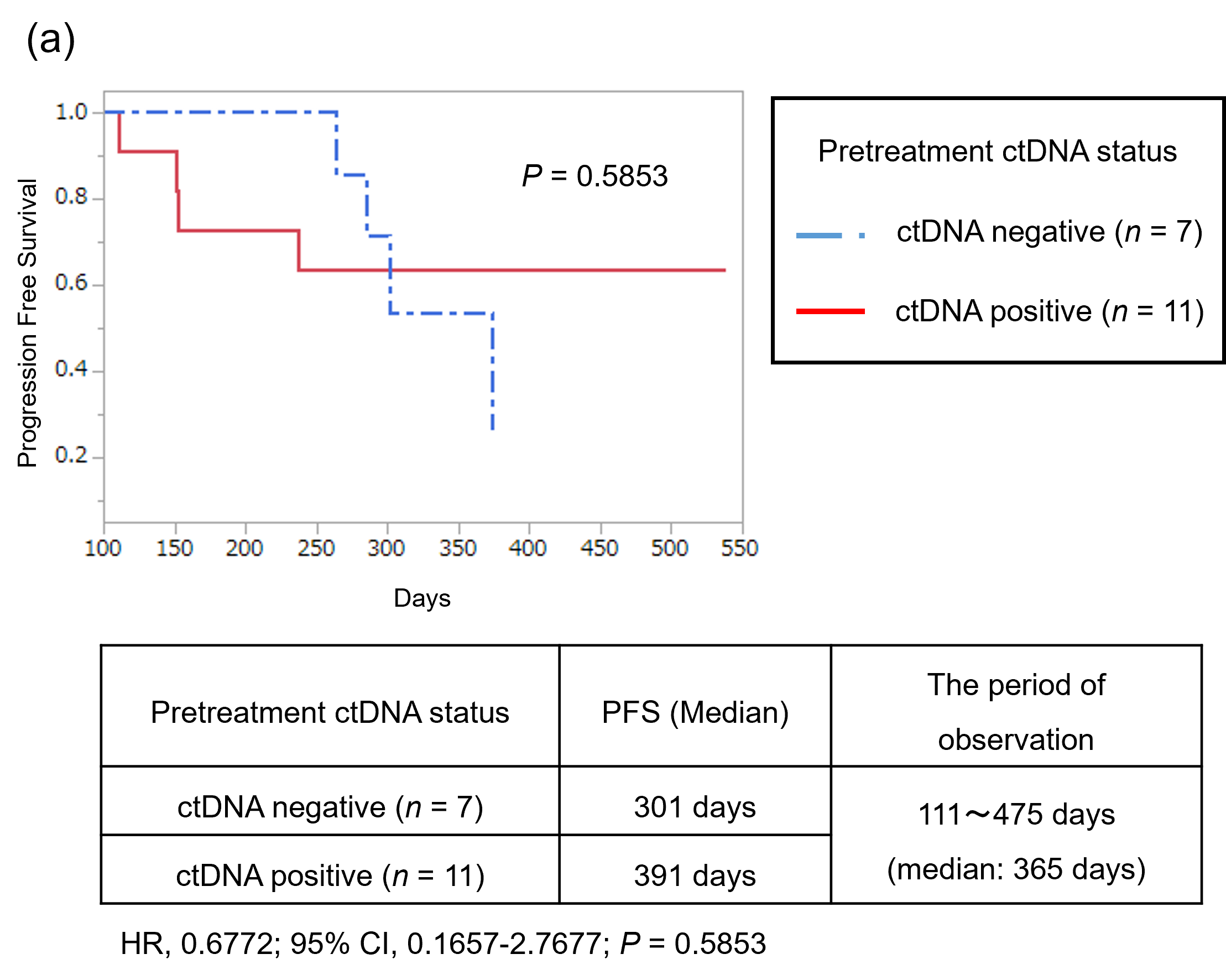

Supplement: Supplementary file 4 — Supplementary file4 (TIF 556 KB) [file 432_2023_5594_MOESM4_ESM.tif]

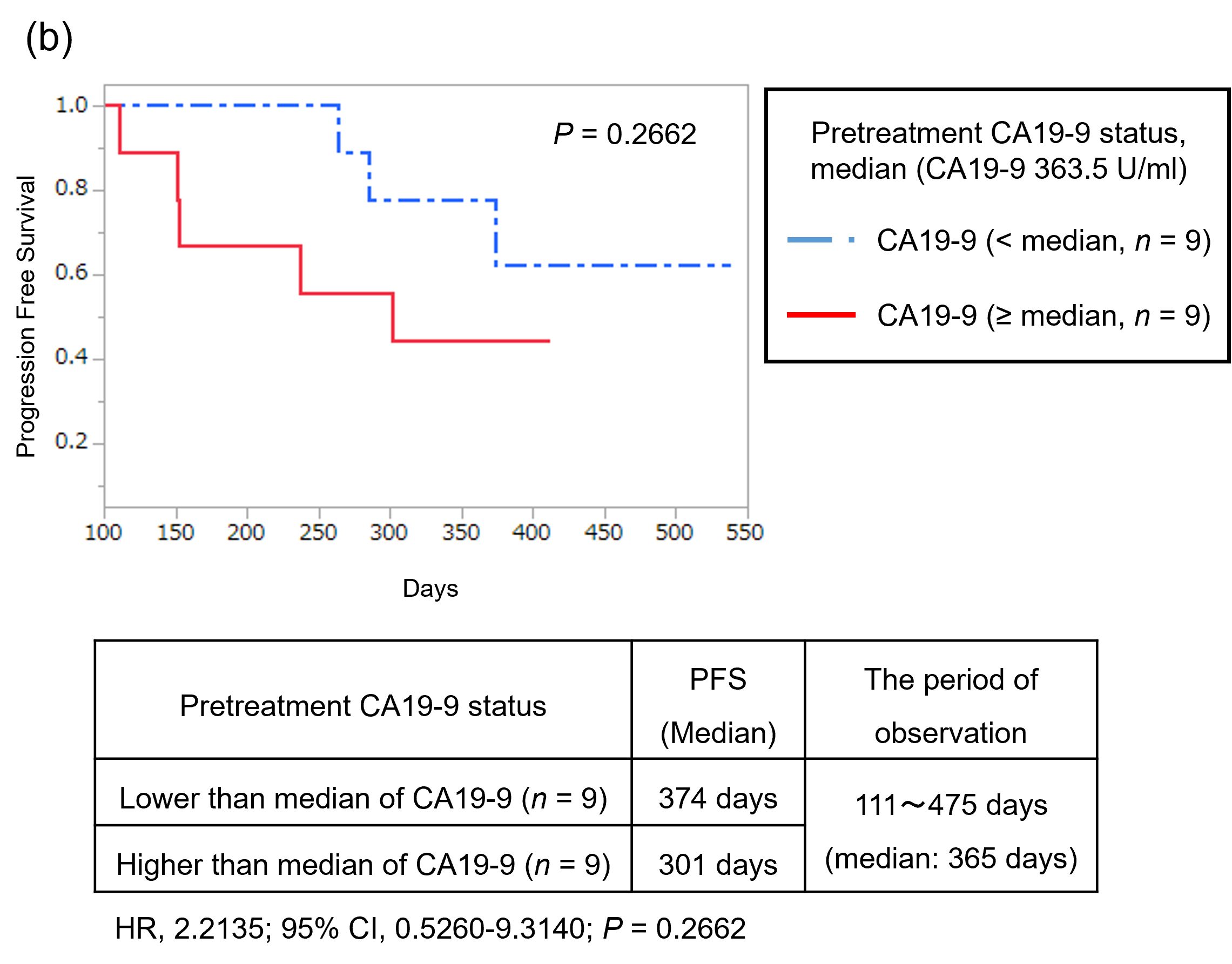

Supplement: Supplementary file 5 — Supplementary file5 (TIF 581 KB) [file 432_2023_5594_MOESM5_ESM.tif]

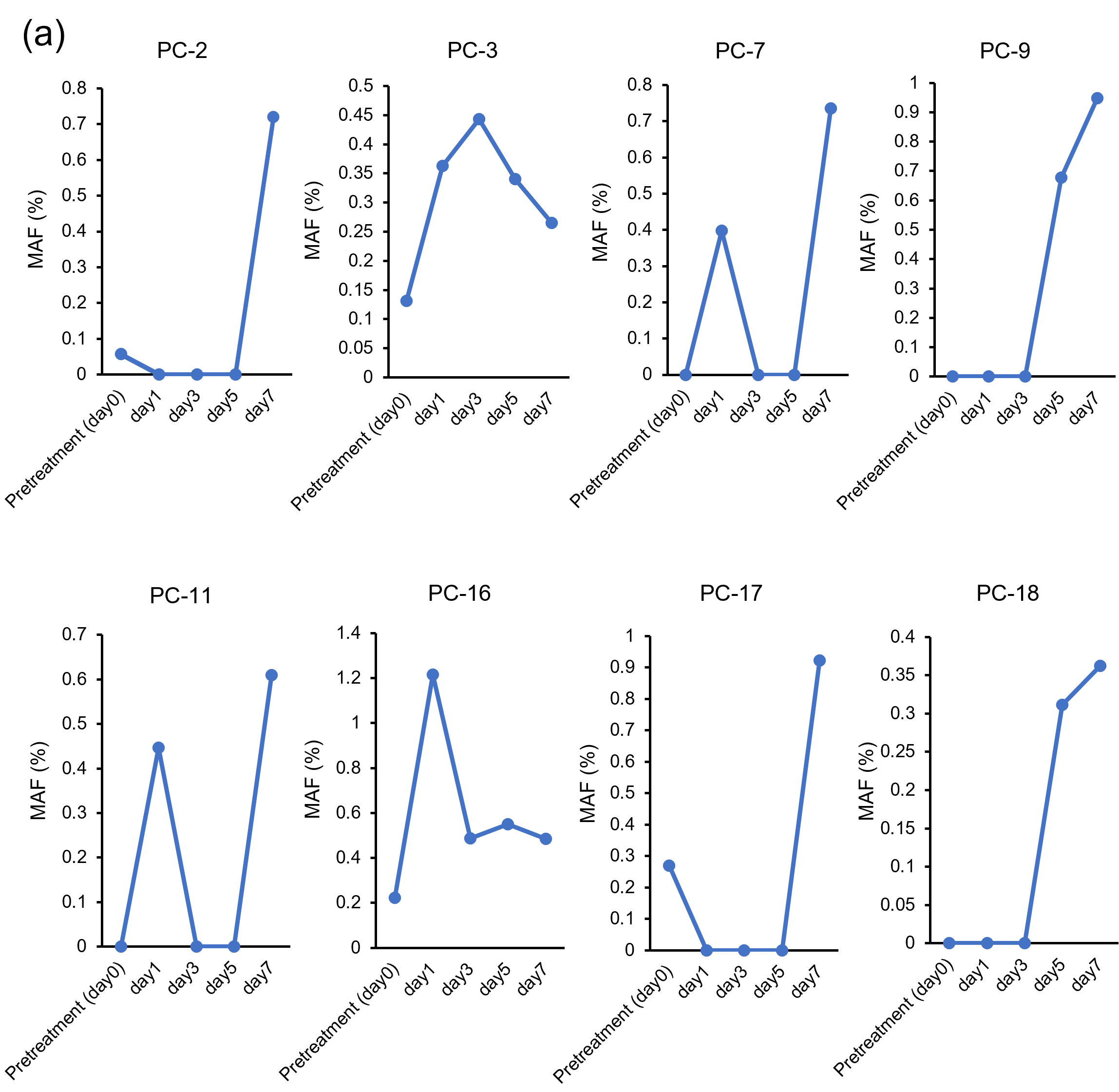

Supplement: Supplementary file 6 — Supplementary file6 (TIF 641 KB) [file 432_2023_5594_MOESM6_ESM.tif]

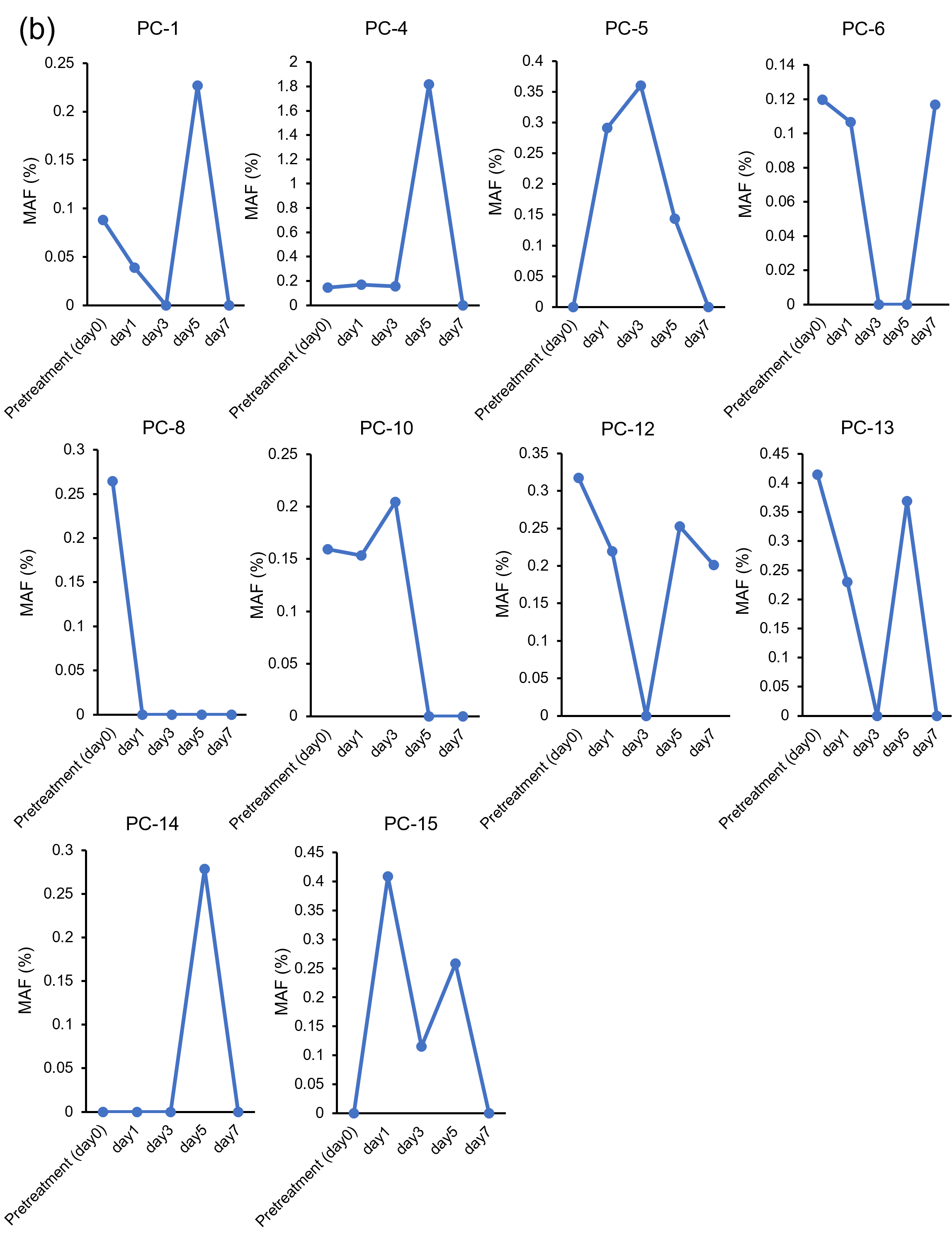

Supplement: Supplementary file 7 — Supplementary file7 (TIF 883 KB) [file 432_2023_5594_MOESM7_ESM.tif]
